# Liver-Directed Locoregional Therapies for Neuroendocrine Liver Metastases: Recent Advances and Management

**DOI:** 10.3390/curroncol31040154

**Published:** 2024-04-05

**Authors:** Cody R. Criss, Mina S. Makary

**Affiliations:** 1 Department of Internal Medicine, OhioHealth Riverside Methodist Hospital, Columbus, OH 43214, USA; cc917811@ohio.edu; 2 Division of Interventional Radiology, Department of Radiology, The Ohio State University Wexner Medical Center, Columbus, OH 43240, USA

**Keywords:** transarterial chemoembolization, embolization, radioembolization, locoregional therapies, neuroendocrine tumor

## Abstract

Neuroendocrine tumors (NETs) are a heterogeneous class of cancers, predominately occurring in the gastroenteropancreatic system, which pose a growing health concern with a significant rise in incidence over the past four decades. Emerging from neuroendocrine cells, these tumors often elicit paraneoplastic syndromes such as carcinoid syndrome, which can manifest as a constellation of symptoms significantly impacting patients’ quality of life. The prognosis of NETs is influenced by their tendency for metastasis, especially in cases involving the liver, where the estimated 5-year survival is between 20 and 40%. Although surgical resection remains the preferred curative option, challenges emerge in cases of neuroendocrine tumors with liver metastasis (NELM) with multifocal lobar involvement, and many patients may not meet the criteria for surgery. Thus, minimally invasive and non-surgical treatments, such as locoregional therapies, have surfaced. Overall, these approaches aim to prioritize symptom relief and aid in overall tumor control. This review examines locoregional therapies, encompassing catheter-driven procedures, ablative techniques, and radioembolization therapies. These interventions play a pivotal role in enhancing progression-free survival and managing hormonal symptoms, contributing to the dynamic landscape of evolving NELM treatment. This review meticulously explores each modality, presenting the current state of the literature on their utilization and efficacy in addressing NELM.

## 1. Introduction

Neuroendocrine tumors (NETs) constitute a heterogeneous group of neoplasms that may occur across various organs, originating from neuroendocrine cells. The current prevalence of NETs is approximately 170,000 and yet the incidence has risen 5–6 fold over the last four decades [1,2]. Clinical management of NETs can be challenging and depends on the primary tumor site, symptom severity, and proliferative activity [3]. NETs can induce paraneoplastic syndromes caused by the release of serotonin, also referred to as “carcinoid syndrome”. According to estimates, approximately 19% of patients can report carcinoid syndrome, which encompasses a myriad of symptoms that include flushing, diarrhea, and wheezing [4,5]. Many of these symptoms have been thought to be mediated by an overproduction of serotonin [5], although other mediators, including serotonin precursors, prostaglandins, tachykinins, and histamine, have also been reported [6].

A distinctive hallmark that influences the prognosis and clinical management of NETs is their propensity for metastasis, which drastically reduces survival [7,8]. For example, the localized disease is typically more indolent with slow-growing lesions and a good prognosis, with reported overall survival (OS) > 30 years [1]. However, metastasis to the liver carries a poor 5-year survival rate, ranging from approximately 19–38%, and the extent of hepatic tumor burden is a crucial prognostic determinant [9,10]. Approximately two-thirds of NETs occur across the gastroenteropancreatic (GEP-NET) system, which constitutes a more aggressive form associated with a worse prognosis [11]. According to the European Neuroendocrine Tumor Society (ENETS) and the North American Neuroendocrine Society (NANETS), for patients with neuroendocrine disease with liver metastases, surgical resection remains the gold standard in the pursuit of curative intent [12]. However, in recent years, there has been a notable emergence of innovative treatment modalities, which may not aim to achieve a cure, but focus on alleviating symptoms, prolonging survival, and improving quality of life [9,13]. Within the battery of treatment approaches for NELM, non-surgical methods including catheter-driven and percutaneous approaches have emerged as adjuncts or viable alternatives to surgery [14]. In the present review, we discuss locoregional therapy approaches for NELM, highlighting the current evidence for their use as well as future areas of investigation.

## 2. Tumor Characterization and Guidelines for Locoregional Therapy 

### 2.1. Diagnostic Imaging and NET Classification

One of the mainstay diagnostic modalities for NET is somatostatin receptor scintigraphy, providing highly sensitive visualization of tumor cells that express somatostatin receptors permitting the identification of primary tumor site as well as metastases. Initially, radiolabeled somatostatin analog octreoscan^®^ was commonly used, but newer radiopharmaceuticals like DOTATOC and DOTANOC are now preferred due to their superior sensitivity (90–94%) and specificity (90–92%) [15,16]. Furthermore, positive emission topography or PET may be combined with computer topography or CT (68 Ga-DOTATOC PET/CT), which has become the gold standard method for the diagnosis and management of NET [17]. For patients with negative PET results and high plasma serotonin levels, other tracers like 18 F-dihydroxy-phenyl-alanine and 11 C-hydroxy-tryptophan can be used [18]. CT and MRI offer superior spatial resolution when compared to PET, and are well suited for identifying small liver lesions, monitoring tumor growth progression, or identifying anatomical variants to better inform locoregional therapy approaches [16]. Furthermore, CT can be used to evaluate tumor treatment response via the radiographic response evaluation criteria in solid tumors (RECIST) or modified criteria (mRECIST), which were initially designed to evaluate anatomical treatment responses geared toward hepatocellular carcinoma. Diffusion-weighted MR imaging also serves as a valuable tool for evaluating NETs by assessing tumor microenvironment and tumor viability, which are altered in regions of necrosis [19]. Alterations in viable tumor regions often coincide with an absence of contrast enhancement, a potential imaging biomarker shown to be indicative of OS in those receiving transarterial locoregional therapy [19].

After the diagnosis of NETs, treatment strategies are frequently guided by disease classification and grade. Based on the WHO 2022 classification, which accounts for proliferation index (Ki-67) and mitotic information, NETs are categorized into well-differentiated neuroendocrine tumor (NET) grades G1, G2, and G3, and poorly differentiated small and large cell neuroendocrine carcinoma [20]. G1/G2 NETs exhibit high expression of somatostatin receptors on their cell membrane (80–95%) [21]. However, somatostatin receptor-based PET/CT is highly sensitive in detecting G1/G2 but G2 may be less sensitive in tumors with higher Ki-6 [21].

### 2.2. General Overview of Treatment Approaches

ENETS and NANETS guidelines support the use of surgical resection as the mainstay curative treatment for liver metastasis [12]. Eligibility for surgical intervention, however, is predominantly contingent upon the extent of the hepatic disease burden borne by the patient. One proposed classification system distinguishes NELM liver distribution by tumor distribution including a single, unilobular lesion (Type 1) or bilobular lesions with a predominant lobe affected with smaller satellite lesions in the other lobe (Type 2), and, finally, multifocal diffuse bilobular disease (Type 3) [22]. Surgical resection may be employed to address the disease with unilobular liver metastasis or, in certain instances, patients with limited bilobular or Type 1 metastasis [14]. Five-year survival after resection in patients with NELM is reportedly 60–75% [23,24]. Given that a considerable number of individuals have multifocal hepatic involvement, achieving complete R0 resection can be challenging, and reported curative resection is only possible in 10–25% of patients, with recurrence at a substantial 50–95% of cases [25,26]. Therefore, in these patients, surgical debulking or cytoreduction is used, with the overall aim of bridging, controlling symptoms, and improving survival [25]. Liver transplantation may also be used as a curative intent treatment for unresectable NELM [27]. ENETS published criteria for patient eligibility for liver transplantation, which include well-differentiated low-grade disease and the absence of extrahepatic disease [28]. However, given that extrahepatic disease is often prevalent in cases of NELM, coupled with global organ shortages and a significant proportion of patients failing to meet surgical eligibility criteria, clinicians are compelled to explore alternative therapeutic options [26]. Hence, for individuals with multiple or extensive metastatic liver lesions, especially in cases where primary resection is constrained (Type 2 and 3), either systemic pharmacological or liver-directed locoregional therapies can serve as viable alternatives to surgery, by extending survival while reducing symptoms and tumor burden [25,28,29].

### 2.3. Alternatives to Surgery

Pharmacological treatment of unresectable NELM includes somatostatin analogs (SSA), such as octreotide and lanreotide [30]. A substantial portion of NETs express somatostatin receptors, with up to 90% of cases for certain subtypes [31]. SSAs work by mimicking the action of somatostatin, a hormone that inhibits the release of various other hormones, including growth hormone and insulin, by binding to somatostatin receptors [32]. Somatostatin signaling is also important for cell growth and apoptosis, and therefore octreotide and lanreotide have been shown to be effective not only at hormonal secretion control but also in immunomodulation and tumor cell angiogenesis inhibition [33,34]. For example, in double-blinded placebo-controlled clinical trials, such as the PROMID and CLARINET studies, patients with NETs treated with octreotide or lanreotide showed prolonged time to progression and progression-free survival (PFS) benefit for NETs, respectively [33,34]. Peptide Receptor Radionuclide Therapy (PRRT) is a relatively new targeted therapy used for unresectable G1/G2 NELM. PRRT involves radioactive substances that are conjugated to tumor cells with somatostatin receptors. PRRT can be performed using either ^90^Yttrium or ^177^Lutetium, bound to chelating agent DOTA and SSA. NETTER-1 phase III clinical trial showed PRRT, ^177^Lu-DOTATATE, has fairly low toxicity with good tumor control for NETs [35,36]. More specifically, ^177^Lu-DOTATATE showed a significantly longer PFS (28.4 months) compared to its control arm (8.5 months), which included long-acting octreotide. Median OS was also reported, with the PRRT arm showing a median OS of 48 months and 36.3 months for the control arm [37]. Although this was an 11.3-month difference between both treatment arms, this was not found to be statistically significant. Current guidelines, namely NANETS and ENETS, recommend the use of PRRT only after the progression of disease while on SSAs [12,38,39,40,41]. Ongoing trials such as NETTER-2 seek to compare ^177^Lu-DOTATATE as a first-line agent with long-acting octreotide for high-grade G2 and G3 NETs [42]. Furthermore, the COMPETE trial aims to compare the outcomes of ^177^Lu-Edotreotide with the M-TOR inhibitor, everolimus, in patients with G2 and G3 gastrointestinal and pancreatic NETs [43]. Some investigations have also explored the use of PRRT in combination with chemotherapy [44].

Liver-directed locoregional therapies include a diverse group of modalities that may be used in diffuse NELM or disease where resection is not possible. Under certain guidelines, they are used as a second line to SSA or systematic therapy. However, in unresectable NETs with metastases isolated to the liver, locoregional therapies may be preferred. Yet, limited data exist to compare the selection of locoregional therapies over systemic agents for unresectable NELM. Investigations have attempted to explore the use of locoregional therapy in combination with other systemic therapies or as part of a combined approach with surgery. Overall, locoregional therapies serve the dual purpose of controlling tumor progression and managing symptomatic manifestations. In fact, a retrospective investigation of patients with NELM explored the use of surgical vs. non-surgical treatment, finding no difference in survival between both management approaches [45]. The following section will explore the application of non-surgical locoregional therapies for NELM by providing a contextualization of the suitability of each modality and elaborating on the underlying rationale guiding their application within the treatment paradigm.

## 3. Locoregional Therapies

Liver-directed locoregional therapies encompass a diverse array of treatment modalities that specifically target tumors and often represent a minimally invasive alternative to surgery [46,47,48]. Much of the initial literature encompassing locoregional therapies has focused on the treatment of primary liver cancers and has since been established as one of the mainstay treatment approaches according to the Barcelona Clinic Liver Cancer guidelines for treating hepatocellular carcinoma [49,50]. In NELM cases where patients are ineligible for hepatic resection due to factors such as compromised performance status, liver-directed locoregional therapies offer valuable options to improve PFS and OS [26]. Typically, locoregional therapies are employed for G1/G2 NELM for controlling larger or multifocal lesions in oligometastatic NELM or for hormonal symptom relief [14]. Furthermore, guidelines such as the European Society of Medical Oncology (ESMO) recommend the use of locoregional regional therapies early in the treatment algorithm for functional tumors to prevent complications such as carcinoid crisis [51]. NANETS also recommends the use of locoregional therapies or chemotherapy for symptomatic NELM over SSAs [38,52]. For G3 NET, systematic chemotherapy is generally recommended [53]. However, NANET guidelines still support the use of liver-directed therapy for G3 NET. However, the decision of when to initiate embolization remains a matter of debate. In fact, a systemic review of non-surgical treatments for pancreatic NELM found limited evidence to support systemic vs. locoregional therapy. Optimal management of unresectable NELM therefore requires a multi-disciplinary approach and is patient-specific. In many cases, it is reasonable to consider liver-directed locoregional therapies as a salvage approach when the liver burden ranges between 10% and 90% [54]. Similarly, NANETS recommends the use of transarterial locoregional therapy approaches with liver-dominant bulky disease with intact liver function [55]. Therefore, the extent of liver involvement using diagnostic imaging can be essential for determining whether locoregional therapies may be effective. In some cases, locoregional therapies such as ablation can be used in combination with surgery to assist with debulking without impacting recurrence rate, survival or post-operative complications [28]. The subsequent section offers a more comprehensive review of each locoregional therapy modality, delineating their respective indications and long-term outcomes. Refer to Table 1 for an overview of descriptions and outcomes of locoregional therapies for NELM.

### 3.1. Transarterial “Bland” Embolization

**Figure 1:**
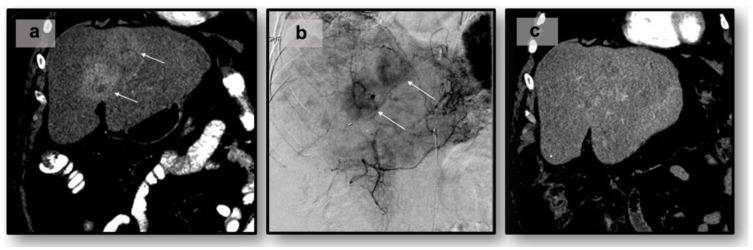
Transarterial chemoembolization of 55-year-old with liver-dominant metastatic NET of small bowel origin to the liver: (**a**) pre-procedure CT. White arrows indicate lesion(s): (**b**) intraprocedural angiogram, (**c**) post-procedural CT.

The rationale for catheter-driven locoregional therapies stems from the observation that NELM tumors typically exhibit hypervascularity (e.g., as evidenced by homogenous or peripheral arterial-phase image enhancement on CT/MRI), primarily deriving their blood supply from the hepatic artery [56]. In contrast to NELM tumor cells, normal hepatic parenchyma predominantly receives its blood supply from the portal vein. In many cases of multifocal bilobar disease, transarterial catheter-driven therapies are recommended for gastroenteropancreatic NET or symptomatic patients [13,57]. Transarterial Embolization (TAE) or “bland” embolization is the selective blockage of blood vessels supplying vascularized NELM tumors, leading to ischemia and subsequent tumor necrosis with minimal damage to normal liver parenchyma. The procedure is accomplished via ultrasound-guided transfemoral or transradial access using an 18–21 gauge needle. Once arterial access via a microcatheter is established, cannulation of the hepatic arteries using a 5 Fr or 6 Fr catheter is performed, followed by angiography to delineate the primary and collateral vasculature [58]. Ultimately, arteries suppling the tumor are injected with embolic agents, such as polyvinyl alcohol, gel foam particles, cyanoacrylate, and microspheres [59]. Follow-up CT in 3 to 6 months can be used to monitor tumor response via the RECIST 1.1 or mRECIST criteria (Figure 1). Transarterial therapies are typically administered at one liver lobe at a time and may require multiple sessions or staged sub-lobar treatments if a large tumor burden is present.

### 3.2. Transarterial Chemoembolization

In contrast to TAE, transarterial chemoembolization (TACE) combines embolization with the infusion of chemotherapeutic agents, such as doxorubicin, cisplatin, mitomycin-C or streptozocin, directly into the tumor-feeding vessels [47,60,61]. This dual approach enhances the local cytotoxic effects while simultaneously inducing ischemia (Figure 1). TACE can be executed conventionally, using an emulsion of chemotherapy and lipiodol (cTACE), or by employing chemotherapy drug-eluting beads (DEB-TACE) [61,62]. Similar to TAE, TACE can also cause post-embolization syndrome. Serious adverse events have been reported in 2–6% of patients receiving TACE [63,64].

TACE has been an effective modality aiming at symptom control, with reported symptomatic response in 78.7% of patients receiving cTACE and 50% receiving DEB-TACE [62]. A comprehensive investigation conducted by Touloupas et al. evaluated the efficacy of TACE in 202 patients who underwent TACE treatment for NELM, reporting a median OS as high as 5.3 years (95% CI 4.2–6.7) [65]. However, other retrospective studies have reported a median OS ranging from 30 to 44 months [66,67,68,69]. Other investigations have also reported the 5-year survival rate of cTACE for NELM to be 28–36% [66,70]. Touloupas et al. further evaluated tumor response via the mRECIST criteria, with authors reporting responders exhibiting a twofold increase in median OS in comparison to non-responders. The survival durations for responders and non-responders were recorded as 80.5 months and 39.6 months, respectively. Interestingly, pancreatic NETs with liver metastasis have shown reduced OS (27.6 months) compared to non-pancreatic NETs (55 months) treated with TACE; however, no differences in PFS have been observed [71]. Of note, negative prognostic factors after TACE treatment include extrahepatic disease, ascites, bilirubin ≥ 2 mg/dL, albumin ≤ 3.5 mg/dL, tumor burden ≥ 70%, and history of receiving three or more trials of systemic therapy [70,72,73]. Interestingly, the neutrophil-to-lymphocyte ratio prior to TACE has demonstrated an association with poorer OS in patients with NELM. This association persists for ratios that remain elevated at 6 months compared to pre-TACE levels [69].

Although some investigations have shown benefits of DEB-TACE over cTACE for the treatment of hepatocellular carcinoma, cTACE has emerged to show improved symptomatic response, as well as OS and progression-free survival compared to DEB-TACE for NELM [62,70]. Yet, DEB-TACE was proposed to be beneficial for patients with poor liver function, as it yields lower liver enzyme elevations and incidence of postembolization syndrome compared to cTACE [62]. In a recent development, the DEB-TACE treatment arm of the Randomized Embolization Trial for Neuroendocrine Tumor Metastases to the Liver (RETNET Trial), which aimed to compare cTACE, DEB-TACE, and TAE, was halted due to a notable rise in hepatobiliary complications, prompting concerns about safety and sparking a nationwide debate [74]. However, since this trial, a recent study showed DEB-TACE to be tolerable with a safe toxicity profile. Albeit retrospective, 87 patients undergoing DEB-TACE for NELM found a complication rate of 2.6%, which included an elevation in liver enzymes that remained stable at 1-month follow-up [75]. Nevertheless, since the RETNET trial, many institutions initially offering DEB-TACE transitioned to prioritizing TAE for NELM.

Limited research, primarily retrospective, has explored the comparison between TAE and TACE for NELM [76]. Overall, TACE and TAE have yielded similar overall and progression-free survival outcomes, as well as tumor and symptomatic responses [77,78]. Some studies observed a tendency of greater improvement in tumor burden and symptom alleviation with TAE over TACE, although without statistically significant distinctions and similar patient tolerance across both methods [77,79].

### 3.3. Important Considerations with Transarterial Approaches

The most frequent adverse event of transarterial approaches includes postembolization syndrome, where patients may experience a combination of symptoms such as abdominal pain, low-grade fever, and nausea/vomiting [80]. The utilization of local analgesia, antiemetics, or preprocedural steroids, either individually or in combination, seems to alleviate this side effect [81]. Complications of TAE include acute cholecystitis, bile duct injury, liver failure, and liver abscess [82].

Patient selection is an important factor to consider in order to mitigate procedural complications. Widely used laboratory criteria for eligibility include serum creatinine < 2, lactate dehydrogenase < 425 mU/mL, aspartate transaminase < 100 mL, platelet count > 100,000/mL, serum bilirubin < 2 mg/dL, and tumor burden <  50% of the liver [83,84]. In addition to the above criteria, other important contraindications of TAE/TACE include portal vein occlusion, biliary anastomoses, poor liver reserve, history of pancreaticoduodenectomy, and poor left ventricular ejection fraction if utilizing TACE with doxorubicin [85,86,87]. Periprocedural carcinoid crisis is also a potentially lethal complication of interventions directed at NETs (e.g., locoregional therapies or surgery) where instrumentation may lead to the release of large quantities of vasoactive compounds leading to hemodynamic instability. Although no universal protocols exist, guidelines such as ENET and NANETs generally recommend the use of perioperative octreotide prophylactically. Generally, boluses of approximately 50–1100 µg can be used with infusions of 50–100 µg/hour [88,89,90]. Radioembolization, on the other hand, has shown some concern over its increased risk of long-term hepatotoxicity [91,92]. However, much of the evidence illustrating hepatoxicity originated from small retrospective investigations, which have not translated into prospective studies [93,94,95]. However, it should be noted that many of these concerns originated from retrospective investigations utilizing supratherapeutic doses.

**Table 1:** Liver-directed locoregional therapy highlights and outcomes.

Locoregional Therapy	Approach	Highlights and Outcomes
Transarterial “bland” Embolization (TAE)	Selective catheterization using embolic agents (e.g., microspheres or gelatin sponge particles) of the hepatic artery and embolization of vessels supplying the tumor	– Advantageous for diffuse, bilobar unresectable disease [54] – Lower risk of post-embolic syndrome than TACE [77]
Transarterial Chemoembolization (TACE)	Selective catheterization using chemotherapeutic agents (e.g., doxorubicin or cisplatin) injected into the hepatic artery supplying the tumor	– Advantageous for diffuse, bilobar unresectable disease [54] – Similar survival outcomes and symptom control to TAE [77,78]
		– DEB-TACE associated with greater hepatobiliary complications than TAE or cTACE [74]
Transarterial Radioembolization (TARE)	Microspheres are loaded with a radioactive isotope (e.g., yttrium-90) resulting in localized radiation therapy to the tumor	– Mixed PFS and OS outcome results compared to cTACE [64,70] – Useful in liver metastasis with fewer, bulky lesions [54] – Preferred over TACE with history of hepatobiliary instrumentation or rapid disease progression
Ablation (e.g., microwave or radiofrequency)	Intra-operative or percutaneous probe(s) employing microwave energy or high-frequency alternativity currents to induce coagulative necrosis of the tumor	– Debulking and cytoreduction, commonly combined with surgery [96] – Liver involvement with less than three lesions measuring ≤ 3 cm, or a single lesion < 5 cm [73]

### 3.4. Transarterial Radioembolization

Transarterial Radioembolization (TARE), or selective internal radiation therapy, involves the delivery of radioactive microspheres directly into the blood vessels supplying the tumor [97,98,99]. Microspheres are typically composed of biocompatible materials like resin or glass, encapsulating the beta-emitting radioactive isotope Yttrium-90, strategically delivered within small blood vessels supplying the tumor. This approach results in localized radiation emission, facilitating precise destruction of the targeted tumor [100]. Resin and glass particles act as carriers for 90Y, both surpassing the diameter of liver capillaries. Resin particles, ranging from 20 µ to 60 µ in diameter, carry an activity of 50 Bq per particle [76,98]. In contrast, glass particles, with a diameter range of 20 to 30 µm, have a higher activity per particle at 2500 Bq [76,98]. This vectorization process ensures the precise delivery of therapeutic radiation to the targeted tumor site during radioembolization procedures [101]. Resin microspheres contain Yttrium-90 (90Y) on their surface, while glass microspheres contain it internally. Despite a similar unit of activity, the fewer glass microspheres, each carrying more activity, contribute to less uniform irradiation, minimizing toxicity and enabling a higher tolerable absorbed dose compared to the more numerous resin microspheres [101,102]. Similar to other transarterial catheter-driven techniques such as TACE and TAE, radioembolization is commonly used in diffuse NELM disease and is typically reserved as a salvage treatment approach [95]. On the other hand, TARE can be completed after one session, but TACE/TAE may require multiple sessions. Helmberger et al. and Wong et al. reported study outcomes for patients with NELM treated with TACE compared to radioembolization with resin microspheres, with an estimated OS of 33 months [91,103]. The investigation by Wong et al. also reported a median PFS of 25 months along with a 3-year PFS rate of 35%. Concurrent meta-analyses of retrospective studies reported a median OS of 28–32 months with a 3-year PFS rate ranging from 45 to 50% [104,105].

### 3.5. Transarterial Radioembolization vs. Chemoembolization

Interestingly, Minh et al. showed cTACE to have superior survival outcomes compared with radioembolization (33.8 months vs. 23.4 months, respectively) [70]. This is not without disagreement, however, where Egger et al. showed similar OS in 248 patients with NELM between TACE and radioembolization [64], although TACE exhibited an overall improved disease control rate. However, meta-analyses encompassing six cohort studies revealed a higher overall survival in TACE when compared to radioembolization [106]. Yet, it is important to note that survival ranges exhibited significant variability in both treatment modalities (TACE 16.8 to 81.9 months; radioembolization 14.5 to 66.8 months). Serious adverse events for radioembolization for NELM are low, and not significantly different from TACE, with reported incidence < 10% [64].

Despite differences in survival outcomes among studies, prognostic factors or clinical scenarios may implicate the use of radioembolization over TACE. For example, prognostic factors such as a Ki67 score are shown to predict different treatment responses to radioembolization or TACE/TAE. Ki-67 is a prominent marker of cell miotic proliferation and is an important factor for categorizing NET tumor grade [107,108]. The Ki-67 score ≥ 3% predicted greater response after radioembolization, while the Ki-67 score < 3% predicted greater benefit with TACE [107]. Additionally, some investigations have suggested that radioembolization may be a sufficient second-line agent for the systemic treatment of diffuse NELM [109]. Interestingly, although extrahepatic disease is associated with poorer prognosis in patients receiving TACE, radioembolization as a second-line treatment does not appear to be affected by extrahepatic disease in terms of OS [67,70,109]. Merits for preferring radioembolization over TACE may also depend on specific clinical scenarios, such as cases with uneven distribution of bulky disease in a single lobe or when the disease is progressing rapidly [54]. This preference arises from apprehension of potential progression outside the treatment field during sequential TACE/TAE. Furthermore, radioembolization may be better suited for patients with bilidigestive anastomosis or a history of Whipple resection, given that TACE/TAE carry a greater risk of biliary ischemia, which can also become infected by gastrointestinal bacteria due to the lack of a physical barrier, facilitating retrograde colonization [54,95,110].

## 4. Ablative Therapies

Ablation may include either radiofrequency ablation (RFA) and microwave ablation (MWA), which involves the insertion of one or more probes directly into the tumor, either percutaneously or laparoscopically, utilizing thermal energy to induce coagulative necrosis, ultimately dismantling malignant cells [111,112,113]. RFA involves the application of high-frequency alternating current, generating thermal energy within the tumor tissue [114]. This hyperthermic environment induces coagulative necrosis, effectively causing denaturation of cellular proteins. RFA is suitable for smaller lesions, offering precise and controlled ablation with minimal impact on the adjacent normal liver parenchyma [73,115]. Additionally, real-time imaging guidance, such as ultrasound or CT, enhances the accuracy of electrode placement, ensuring optimal treatment outcomes. On the other hand, MWA employs electromagnetic waves to generate heat within the tumor tissue, resulting in thermal injury and cellular destruction [111,116]. Compared to RFA, microwave ablation may offer faster and larger, more homogeneous tissue heating, potentially improving treatment efficacy for larger or irregularly shaped lesions. The ability of microwaves to penetrate tissues with less susceptibility to the “heat-sink” effect is a notable advantage, making it a viable option for neuroendocrine liver metastases in challenging anatomical locations [117,118,119]. Most of the literature reporting ablative approaches to NELM encompasses the use of RFA or MWA. However, other ablation techniques also include percutaneous ethanol injection, which is a highly hydrophilic and cytotoxic agent, or cryoablation, which involves the controlled application of extremely low temperatures to induce cell death destruction through both direct freezing and microvascular disruption [47,120]. However, few investigations have explored the use of these methods and the scientific literature has been subjected to mostly small retrospective studies or case reports compared to other ablative methods [121,122].

According to current guidelines, vascular and ablative locoregional treatments are recommended exclusively for G1-G2 NETs when there are metastases primarily affecting the liver, and the extrahepatic disease remains stable. Ablative techniques are exclusively used for limited or oligometastatic liver disease, including scenarios with less than three lesions measuring ≤ 3 cm, or a single lesion < 5 cm, but may also be considered in conjunction with hepatic resection [73]. Interestingly, Perodin et al. investigated outcomes when microwave ablation is used as an alternative to surgical management for NELM, reporting a lower incidence of both minor and major complications when compared to surgery [123]. No significant disparities were observed in terms of local recurrence or mean survival between the two treatment modalities. Furthermore, a prospective investigation utilized ablation under laparoscopic guidance for NELM, reporting a median OS of 3.9 years after receiving RFA [124]. In this report, liver lesions with diameters greater than 3 cm predicted worsening survival.

Similar to other liver-directed locoregional therapies, ablation is commonly employed in a multimodal approach. For example, ablation may be used to reduce unresectable lesions or to circumvent the need for extensive liver resection [28]. Ablation as a debulking or cytoreductive approach is typically tailored for diseases where >70–90% cytoreduction can be achieved, which equates to prolonged OS and PFS [125]. When used as an adjuvant to surgery, overall survival has been reported to be 80% and 59% for 5 and 10 years, respectively [96]. A systematic review of ablation utilization in NELM revealed a 92% improvement in symptoms following RFA, with a median duration of symptom relief spanning 14 to 27 months [126,127]. In the case of multifocal, unresectable disease where systemic or medical therapy is warranted, ablation can also be used in the interim to delay disease progression. For example, a small retrospective study showed prolonged PFS in 88% of patients with NELM after receiving ablation, reporting a median PFS of almost 16 months prior to initiating systematic therapy [128]. MWA is less susceptible to the heat-sink effect when compared to RFA and may be useful in lesions > 4–6 cm due to larger ablation zones [129]. A retrospective investigation showed a 77% success rate in NELM treated with operative MWA with or without concomitant resection receiving clinical improvement in 95%of patients after MWA. Furthermore, 5-year OS rates were 70% [127].

## 5. Multimodal Strategies and Future Directions

The implementation of multimodal therapy approaches may harbor improved survival outcomes [130]. For instance, radioembolization has been combined with chemotherapeutic agents such as everolimus and SSA in a small phase 1b investigation including patients with advanced NELM disease, reporting PFS and OS of 18.6 and 46.3 months, respectively [131].

A few reports have utilized a combination of radioembolization with other treatment approaches like PRRT and appears to be safe with promising results [132,133,134]. This regimen may be particularly beneficial in patients with bulky disease or metastatic disease with predominant liver burden, particularly because Lutetium-177 has a reported tissue penetration of 2–4 mm and the reported PFS is worse with larger lesions [135]. A small retrospective investigation of 44 patients illustrated a median OS of 41 months and a 3-month disease control rate of 91% after receiving radioembolization within previous PRRT therapy. Furthermore, when compared to just radioembolization, a comparative study of 27 patients showed a treatment response rate of 86.6% compared to 66.6% for patients treated with PRRT radioembolization compared to radioembolization alone [136]. Differences were not found to be significant, as OS in the combination therapy group was 67.5 months compared to 34.9 months in the radioembolization alone group. Furthermore, a retrospective investigation of 23 NELM cases reported no additive liver toxicity when radioembolization was used with PRRT, ^177^Lu-DOTATATE, with a median follow-up period of 38 months [137]. These results call into question initial concerns of hepatotoxicity associated with radioembolization; however, larger-scale investigations are warranted.

As mentioned previously, the presence of bulky NELM can negatively impact the PFS benefit of PRRT [138]. Hence, studies like the Lutetium Intra-arterial (LUTIA) trial have sought to compare the intra-arterial administration of 77Lu-DOTATATE with conventional intravenous administration in patients with G1/G2 NETs featuring NELM [139]. Patients in this trial were randomly assigned to receive intra-arterial peptide receptor radionuclide therapy (PRRT) in either the left or right hepatic lobe for four consecutive cycles, with the primary endpoint being the tumor-to-non-tumor uptake ratio. Although toxicities remained similar from both administrations, intra-arterial 77Lu-DOTATATE did not yield a clinically significant difference in uptake compared to conventional intravenous administration [140]. While these findings may not be encouraging, some contend that the trial has laid the groundwork for future investigations into intra-arterial PRRT [141]. The explanation for these results could potentially lie in characteristics unique to 77Lu-DOTATATE compared to other forms of PRRT or variations in its commercial administration, which may have introduced confounding factors.

## 6. Conclusions

In conclusion, NETs present an intricate challenge, characterized by a notable surge in prevalence and the emergence of cutting-edge treatment modalities in recent years. The focal point of treatments has shifted towards ameliorating symptoms and augmenting the quality of life for individuals grappling with NELM. Locoregional therapies, inclusive of catheter-driven interventions and percutaneous interventions, assume a pivotal role in the management of NELM, offering alternatives or complementary measures to surgical intervention.

While certain investigations posit potential advantages of TACE/TAE, radioembolization, and ablation, all interventions evince efficacy in enhancing overall survival rates under appropriate clinical circumstances. It is imperative to judiciously factor in patient-specific considerations and response criteria when tailoring treatment strategies. Furthermore, the ongoing exploration of avant-garde approaches underscores the dynamic and evolving landscape characterizing the management of NELM. This comprehensive review illuminates the intricate and multifaceted nature of NELM treatment, showcasing evidence that supports the utilization of locoregional therapies throughout the treatment continuum, especially in cases of unresectable diseases. It underscores the importance of embracing personalized and all-encompassing strategies to attain optimal therapeutic outcomes.
